# Socioeconomic Factors Influencing Self-reported Outcomes After Posterior Wall Fractures of the Acetabulum: Lessons Learned From a Hispanic Population

**DOI:** 10.5435/JAAOSGlobal-D-20-00162

**Published:** 2020-10-01

**Authors:** Manuel Rodríguez-Pérez, Ariel Dávila-Parrilla, Lenny Rivera, Gerardo Olivella, Andrés Muñiz, Norman Ramírez, Luis Lojo-Sojo

**Affiliations:** From the Orthopedic Surgery Department, University of Puerto Rico (UPR) Medical Sciences Campus, San Juan, Puerto Rico (Dr. Rodríguez-Pérez, Dr. Dávila-Parrilla, Dr. Rivera, Dr. Olivella, Dr. Muñiz, and Dr. Lojo-Sojo), and Pediatric Orthopedic Department, Mayagüez Medical Center, Mayagüez, Puerto Rico (Dr. Ramírez).

## Abstract

**Introduction:**

Demographic and socioeconomic factors are important determinants that may affect patient self-reported outcomes after acetabular fracture surgery. Hispanics, as a minority group, have an increased tendency to suffer demographic and socioeconomic disparities. At the present time, there is scant information regarding their role among Hispanic patients with acetabular fractures. The aim of this study was to investigate whether demographic or socioeconomic factors would affect satisfaction and self-reported functional outcomes in Hispanic patients who endured open reduction and internal fixation (ORIF) of posterior wall fractures of the acetabulum.

**Methods:**

A cross-sectional study of 78 patients with posterior wall fracture of the acetabulum treated with ORIF from 2011 to 2017 was performed. Data from demographics (age, sex, and body mass index [BMI]) and socioeconomic factors (household income, highest educational level achieved, employment status, type of medical insurance, workers' compensation involvement, and injury-related litigation process) were obtained and compared with the Short Musculoskeletal Form Assessment-46 (SMFA-46) questionnaire using a multivariable logistic regression analysis.

**Results:**

Unemployment was the most significant variable associated with dissatisfaction among 15 of the 46 responses of the SMFA-46 (*P* < 0.048). Patients who had an overweight or obese BMI and had an age of 41 years or older exhibited significantly worse outcomes in 7 of the 46 questions (*P* < 0.049). Finally, women were less likely to be satisfied in 1 of the 46 questions (*P* = 0.028). No notable difference was observed in any of the SMFA-46 responses regarding insurance plan, educational level, workers' compensation involvement, and injury-related litigation process.

**Discussion:**

A strong association exists between dissatisfaction after ORIF of posterior wall fractures of the acetabulum and employment status, BMI, and age among the Hispanic population. Addressing socioeconomic factors can be critical to overcome dissatisfaction and improve functional status among Hispanic patients with acetabular fractures.

The complexity of the acetabulum and the surgical management of its fractures have led to a historically challenging pursuit of achieving consistent, feasible postsurgical results. Earlier modalities in the treatment of acetabular fractures mainly provided unacceptable results, and affected patients were deemed to endure a severe decline in their quality of life. It was not until the later decades of the 20th century that acceptable outcomes were fundamentally set in motion through the contributions made by Judet et al and Letournel.^1^ To better assess and dictate the choice of approach for the management of these fractures, they developed an anatomic classification system based on five elementary and five associated fracture patterns evaluated through AP and oblique hip radiographs. The elementary fracture types were categorized into isolated posterior wall, posterior column, anterior wall, anterior column, or transverse patterns. Concurrently, the associated fracture types were divided into associated both columns, transverse and posterior wall, T shaped, anterior column or wall and posterior hemitransverse, or posterior column and posterior wall patterns.^2,3^ Among these, isolated posterior wall fracture is the most frequently encountered pattern.^4,5^ Despite being one of the most simple-appearing fracture types, genuine outcomes become apparent only after long-term patient evaluation takes place. Throughout the years, multiple studies have suggested that the best predictor of a long-term satisfactory clinical outcome is an appropriate anatomic reduction of the fracture.^6,7,8^

However, other factors exist that could possibly affect patient outcome even after radiographic evidence of adequate surgically treated posterior wall fractures of the acetabulum. In 2007, Moed and McMichael^7^ demonstrated that although radiologic findings of patients with a posterior wall fracture of the acetabulum were adequate, clinical presentation were markedly worse than normative reference values. Correspondingly, they recommended that research efforts should be directed toward the identification of socioeconomic elements and other underlying determinants that may affect satisfaction and functional outcome, potentially leading to suitable treatment interventions. This statement suggests that patients' premorbid socioeconomic status may be a critical determinant of their emotional response to injury and ultimate functional outcome.^7,9,10^ In other types of orthopaedic disorders, socioeconomic factors such as ethnic differences, low socioeconomic status, and compensation litigation have been reported to be factors that may influence the outcomes after surgery.^11,12,13,14^ Two recent studies exposed that socioeconomic factors substantially affected patients' perceived clinical outcome after total hip and knee arthroplasties, but nothing has been described regarding posterior wall fractures of the acetabulum that underwent open reduction and internal fixation (ORIF).^15,16^ Although emerging attention exists on the impact of socioeconomic elements in the postsurgical outcome, these factors remain empirically questionable in the current literature. Attention to these factors is even more essential nowadays because patients' postsurgical perceptions have revolutionized, now expecting to achieve an idealistic, earlier, and complete recovery.^10^

Therefore, the aim of this study was to investigate whether demographic (age, sex, or body mass index [BMI]) or socioeconomic factors (household income, highest educational level achieved, employment status, type of medical insurance, workers' compensation involvement, or injury-related litigation process) would affect satisfaction and self-reported functional outcomes in Hispanic patients who endured ORIF of posterior wall fractures of the acetabulum. It has been documented that among the USA population, Hispanic patients have a higher prevalence of posttraumatic stress disorder symptoms after musculoskeletal injuries and are also more prone to report lower self-reported outcomes after total knee arthroplasty.^9,17^ Thus, we hypothesized that dissatisfaction and poorer outcomes would be related to unfavorable demographic and socioeconomic factors.

## Methods

A retrospective cross-sectional study of all patients who presented to a single state designated level I trauma center with posterior wall fracture of the acetabulum that required ORIF from 2011 to 2017 was performed. All the surgeries were executed by the same fellowship-trained orthopaedic trauma surgeon. Patients included in the study had (1) a unilateral elementary type posterior wall fracture of the acetabulum as defined by Judet et al and Letournel,^2,3^ (2) 18 years of age or more, (3) a minimum of 2 years of postoperative follow-up, (4) completed the Short Musculoskeletal Form Assessment-46 (SMFA-46) questionnaire,^18^ and (5) radiologic findings with no evidence of marginal impaction, intra-articular fragments or osteonecrosis. Pediatric cases, patients who were either treated nonsurgically or required primary total hip arthroplasty, or had multiple lower extremity injuries or had a delay in reduction, were not included in the study.

Approval from the Institutional Review Board of the Institutional Review Board of the University of Puerto Rico, Medical Sciences Campus, was obtained for this study. The sample data included information on patient demographics, socioeconomic status, mechanism of injury, and comorbidities. Demographics such as age, sex, and BMI were evaluated. Age was categorized in two groups based on the average age of the total sample data, in which the younger group was constituted by those 40 years or younger and the older group was constituted by those 41 years or older. BMI was divided as normal (18.5 to 24.9), overweight (25.0 to 29.9), and obese (30.0 or more).^19^ The socioeconomic data included (1) household income, (2) highest educational level achieved, (3) employment status, (4) type of medical insurance, (5) workers' compensation involvement, and (6) injury-related litigation process. For analysis, household income was grouped as either low-income (less than $20,166 per year) or high-income (more than $20,166 per year) based on the 5-year estimates (2014 to 2018) of the United States Census Bureau, the American Community Survey, and the XX Community Survey of the region of XX.^20^

### Patient Satisfaction and Self-reported Functional Outcome

Outcomes toward patients' self-reported personal satisfaction and function on undergoing surgery was measured through the SMFA-46 questionnaire. The survey was fulfilled at the outpatient clinic at the 2-year postoperative follow-up. Every patient, voluntarily, answered the 46-item questionnaire. This questionnaire elucidates the patient's function (34 questions) and bothersome index of his/her symptoms (12 questions). The SMFA-46 measures self-care, sleep/rest, hand/fine motor skills, mobility, housework, employment/work, leisure/recreation, family relationships, cognition/thinking, and emotional adjustment/coping/adaptation.^18^ Based on a previous study, SMFA-46 responses were grouped into two broad categories as either “never/rarely” (satisfied) or “sometimes/often/frequent” (dissatisfied).^15^ Every response provided in the questionnaire was included and then juxtaposed to demographic and socioeconomic data.

### Statistical Analysis

Statistical analyses were performed using Microsoft Excel and GraphPad Prism eighth version software. Descriptive statistics were used to analyze the frequency of continuous data. Multiple logistic regression with adjusted odds ratio were used to assess the association of categorical demographic and socioeconomic factors with the SMFA-46 questionnaire responses. A *P*-value of 0.05 or lower was considered statistically significant.

## Results

A total of 78 patients who underwent ORIF for a posterior wall fracture of the acetabulum from 2011 to 2017 fulfilled the inclusion criteria. Sixty-one patients were men (61 of 78 = 78.2%), with an average age at initial presentation of 41.4 ± 16.3 years (range 18 to 85) and an average BMI of 28.6 ± 5.4 (range 20 to 45). The most common mechanism of injury was motor vehicle accident in 62 cases (79.5%), followed by fall from more than 10 feet high in 7 cases (9.0%), fall from standing height in 6 cases (7.7%), and pedestrian accident in 3 cases (3.8%). Forty patients (40 of 78 = 51.3%) had medical comorbidities such as high blood pressure (14 pts), type 2 diabetes mellitus (10 pts), bronchial asthma (8 pts), psychiatric disorder (3 pts), heart disease (1 pt), chronic anemia (1 pt), malignancy (1 pt), hypothyroidism (1 pt), and rheumatoid arthritis (1 pt). In addition, 36 patients (36 of 78 = 46.2%) consumed alcohol at least in moderate amounts, and 12 patients were active smokers (12 of 78 = 15.4%).

Patients who completed a college degree were the most predominant population in our study (44 of 78 = 56.4%). Sixty patients (76.9%) had their medical costs covered by private medical insurances such as workers' compensation (9 patients) and motor vehicle accident's compensation (51 patients). Forty-six patients (59.0%) were found to be unemployed. A total of 71 patients (91.0%) had an annual household income less than $20,166. Sixteen patients (20.5%) were involved in an injury-related litigation process.

The prevalence rate of each SMFA-46 questionnaire response by demographic and socioeconomic variables, adjusted and grouped with a multiple logistic regression analysis, is illustrated in Supplemental Table 1 (Supplemental Digital Content 1, http://links.lww.com/JG9/B310). The most prevalent variable associated with lower satisfaction among the SMFA-46 questionnaire was the employment status, with 15 of the 46 responses being significantly different (*P* < 0.048). Patients who had an overweight or obese BMI and had an age of 41 years or older, had significantly worse outcomes in 7 of the 46 questions (*P* < 0.049). Patients with an annual mean household income of more than $20,166 reported they were more likely to be dissatisfied in 2 of the 46 questions (*P* < 0.035). Finally, women were less likely to be satisfied in 1 of the 46 questions (*P* = 0.028). No significant difference was noted in the prevalence of any of the SMFA-46 responses regarding insurance plan, educational level, workers' compensation, and injury-related litigation process (Supplemental Table 1, Supplemental Digital Content 1, http://links.lww.com/JG9/B310).

Evaluation of the prevalence of the questionnaire responses stratified by functional and severity results (questions 1 to 34) revealed that unemployment had the most notable dissatisfying outcomes in 14 of the 34 questions, followed by overweight or obese BMI and age of 41 years or older with 5 of the 34 questions. Interestingly, a stratification analysis of the prevalence of bothersome index (questions 35 to 46) demonstrated that overweight or obese BMI and age of 41 years or older were markedly affected in 2 of 12 questions. Patients who were unemployed only seem to be bothered by only 1 of the 12 questions (Supplemental Table 1, Supplemental Digital Content 1, http://links.lww.com/JG9/B310).

Throughout the questionnaire, multiple factors displayed statistically significant relationships toward a singular outcome measure in a Hispanic population. Patients who were either overweight, obese, or unemployed were more likely to be dissatisfied with getting in or out of a low chair, with getting in and out of a bathtub or shower, and to have complications with bending down or kneeling. Patients who were either 41 years or older, overweight, obese, or had a low annual mean household income were more likely to be dissatisfied with shopping for groceries or other things. Patients who were either 41 years or older, overweight, or obese were more likely to have issuess with walking. Patients who were either 41 years or older or unemployed were more likely to have issues with pivoting and with sexual activities. Finally, patients who were either women or unemployed were more likely to avoid using the painful limb or back (Supplemental Table 1, Supplemental Digital Content 1, http://links.lww.com/JG9/B310).

## Discussion

Despite contemporary superior surgical techniques and quality of implants, unsatisfactory outcomes after surgically treated posterior wall fractures of the acetabulum have been found to approximate 30%.^21,22^ Several clinical risk factors identified include intra-articular comminution of three fragments or more, marginal impaction, weight-bearing dome involvement, femoral head injury, a delay in reduction of a dislocated hip greater than 12 hours, residual fracture gap width greater than or equal to 10 mm after ORIF, and age older than 55 years.^8,21,23^ Despite these identified risk factors, attempts to accurately prognosticate long-term clinical outcomes and the need of arthroplasty surgery after posterior wall fractures of the acetabulum that underwent ORIF have not led to meaningful success, thereby underlying the need for further research.^24^ However, no study has explicitly evaluated the intertwined behavior of patient satisfaction, self-reported functional outcome, and socioeconomic and demographic factors in the postoperative period.

The impact of age and sex on clinical outcomes after acetabular fracture has provided heterogeneous results. However, most of the literature suggest that elderly patients reflect suboptimal outcomes, irrespective of the sex.^25^ By contrast, an elevated BMI has been consistently found to negatively affect outcomes and is an independent risk factor for complications after either surgical or nonsurgical treatment.^26^ Our findings are comparable with previous studies in which sex has limited influence in outcomes, but older and obese patients were more dissatisfied and reported worse functional outcomes. On the other hand, sex seems to be more consistently related to total knee arthroplasty, where women report lower satisfaction than men.^15^

Socioeconomic factors such as low household income may limit access to adequate postoperative treatment, thus increasing the likelihood of poor functional outcomes.^27^ In total shoulder arthroplasty, patients with a lower socioeconomic status not only have limited access to this type of surgery but also have worse preoperative function and pain, higher rates of opioid usage, and an increased risk of developing chronic back pain, and psychological distress.^13^ After total knee and hip arthroplasty, these patients had lower postoperative functional scores, more pain, and reported lower satisfaction.^15,16^ The same pattern was observed in patients who underwent surgical management of nonunion fractures.^14^ On the contrary, in our study population, we found that low household income, independently, was a limited contributor to worse outcomes.

The decision-making process and posttraumatic treatment require thorough discussion and corroboration of patient's understanding of the risks and benefits of undergoing any given treatment. Because patients' degree of comprehension and expectations may be related to their educational level, it is imperative to tailor the decision-making process to his or her unique characteristics. In spine surgery, patients with less than a college degree were found to be related to lower satisfaction scores.^28^ After the evaluation of multiple socioeconomic factors related to negative outcomes after surgical management of nonunion fractures, an even larger influence was evidenced in patients with a low educational level.^14^ By contrast, Chapin et al^29^ did not find a notable relationship between educational level and patient satisfaction and outcomes after spine surgery, after noticing that they were rather markedly influenced by employment and disability status at the time of the surgery. Our data mirror these findings, suggesting that educational level was not an independent risk factor for worse satisfaction and outcomes.

The role of employment status in satisfaction and self-reported functional outcomes has been extensively evaluated in joint arthroplasty surgery.^30^ Unemployment is a considerable risk factor for worse outcomes, becoming more detrimental when patients are unemployed in the 3-month period before surgery.^15^ When employment status is evaluated with ethnicity, expectations are even more negatively attenuated in unemployed minority groups.^31^ Multivariable regression analysis of our results suggests that employment status was the most notable risk factor for worse satisfaction and outcomes. Taking into consideration that the unemployment rate in XX is more than double the unemployment rate in the United States as of 2019, our finding exemplifies what has been reported in joint arthroplasty surgery.^20^

Throughout the orthopaedic surgery field, numerous efforts have been dedicated to increase patient satisfaction, supported by the fact that it is a recognized quality measure by the Center for Medicare and Medicaid Services.^32^ It has been documented that the type of medical insurance, private vs government-issued, can influence whether a patient receive surgical treatment of some orthopaedic disorders. However, limited information exists regarding its impact on satisfaction and outcomes.^33^ Our study did not find a notable relationship regarding the type of medical insurance. This could be due to the nature of the specific disorder evaluated in this study, which could result differently in other chronic or elective disorders.

Work reintegration after acetabular fractures has caused a substantial toll on social systems, with only 69% of patients returning to their former occupational position.^34^ Those patients who receive workers' compensation often do not demonstrate objective clinical findings related to unfavorable clinical results after undergoing total knee arthroplasty, yet there is an increased likelihood to report fair or poor results when compared with those who do not receive workers' compensation.^35^ This tendency was not replicated in our study, which failed to demonstrate any notable difference.

Orthopaedic surgery is among the specialties most involved in medical malpractice claims, increasing considerably during the past 10 years.^36^ Malpractice is defined as “professional responsibility derived from inadequate medical care caused by lack of competence, negligence, or deceit,” whereas medical liability is defined as “obligation to repair or satisfy the consequences of medical action from a penal, civil, or administrative perspective.” ^37^ It has been perceived that the trauma setting predisposes to a litigation process, but a recent study has found that the risk for orthopaedic surgeons who deal with trauma surgery is not greater than that in elective surgery.^38^ It seems that the presence of an injury-related litigation process is not a determinant factor for dissatisfaction after posterior wall fractures of the acetabulum. Consequently, surgeons should not expect worse self-reported outcomes when encountering these patients.

Ethnic disparities in perceptions toward orthopaedic injuries have been limitedly evaluated in the literature. One study that evaluated socioeconomic factors related to functional outcomes after total knee arthroplasty found that minority groups such as Hispanics and African Americans reported inferior results on multiple functional outcome measures.^15^ However, the authors recognize this finding may be interrelated to low mean household income, rendering it difficult to determine whether it was minority status or income that was the most important factor contributing to the inferior results. In musculoskeletal trauma patients, those who were Hispanic women had a documented history of psychiatric diagnosis and were born in the United States and had an increased likelihood of developing posttraumatic stress disorder symptoms.^17^ This increased likelihood may be attributable to acculturation, violence, racism, and limited patient-provider communication manifested as incongruent value systems and cross-cultural miscommunication. The Hispanic population emerged by 15.2 million (43%) between 2000 and 2010, hence representing the fastest growing ethnic population in the United States.^12^ It is expected that by 2060, it will virtually double in size from 18% to 28% of the entire US population.^39^ This progressive tendency reflects the urgent need for comprehensive data on characteristics of the Hispanic patient population.

The retrospective nature of this study cannot be used to demonstrate a cause-and-effect relationship and may confer a limited degree of generalizability to another type of population. Although no larger studies with these types of injuries were reported previously, a larger prospective study group could serve to identify a difference between the groups that we could not identify because of the limited population size. Other factors such as complications could be evaluated in future studies to find more specific associations with outcomes and decrease confounding factors.

In conclusion, our results suggest that dissatisfaction and poorer outcomes are related to unfavorable demographic and socioeconomic factors. Accordingly, a multidisciplinary approach should be adopted when treating Hispanic patients with acetabular fractures. Older, obese, and unemployed patients should be approached by medical staff members who can optimize the preoperative and postoperative protocols to improve outcomes and decrease the risk of these patients enduring dissatisfaction and functional disability. More importantly, appropriate social work evaluation and physical therapy rehabilitation programs should be instituted early to help patients regain their previous employment status because this may be a modifiable variable that can considerably improve outcomes. It is consequently recommended to recognize demographic and socioeconomic factors as potential determinants of patient prognostication and risk stratification.

## Supplementary Material

SUPPLEMENTARY MATERIAL
