# Supplementary material for: Socioeconomic Factors Influencing Self-reported Outcomes After Posterior Wall Fractures of the Acetabulum: Lessons Learned From a Hispanic Population
Source: J Am Acad Orthop Surg Glob Res Rev. 2020 Oct 1;4(10):e20.00162. doi: 10.5435/JAAOSGlobal-D-20-00162 (PMC7537825; doi:10.5435/JAAOSGlobal-D-20-00162)
Supplement: SUPPLEMENTARY MATERIAL [file jagrr-4-e20.00162-s001.docx]

**Supplemental Table 1**: Adjusted odds ratio of SMFA-46 questionnaire responses associated with variables of interest using multiple logistic regression (N = 78)

| **Outcome Measure** | **R^2** | **Age > 41 y/o** | **BMI (18.5 - 24.9)** | **Female Sex** | **Education (Less than College)** | **Unemployed** | **Government Insurance** | **Household Income** | **Workers’ Compensation** | **Injury-Related Litigation Process** |
| --- | --- | --- | --- | --- | --- | --- | --- | --- | --- | --- |
| **Satisfaction Functional Index** | | | | | | | | | | |
| **1. Dissatisfied getting in or out of a low chair** | | | | | | | | | | |
|  | 0.297 | 2.71 (0.83 to 9.70)  (p = 0.107) | 0.11 (0.02 to 0.41)  (p = 0.002) | 0.67 (0.14 to 3.05)  (p = 0.594) | 0.24 (0.04 to 1.03)  (p = 0.071) | 6.83 (1.62 to 38.11)  (p = 0.015) | 0.45 (0.04 to 3.75)  (p = 0.476) | 0.20 (0.01 to 3.30)  (p = 0.312) | 0.27 (0.03 to 1.67)  (p = 0.189) | 0.63 (0.14 to 2.90)  (p = 0.541) |
| **2. Dissatisfied opening medicine bottles or jars*** | | | | | | | | | | |
| **3. Dissatisfied shopping for groceries or other things** | | | | | | | | | | |
|  | 0.277 | 5.72 (1.84 to 19.88)  (p = 0.004) | 0.20 (0.03 to 0.88) (p = 0.049) | 1.47 (0.30 to 6.81)  (p = 0.624) | 2.77 (0.68 to 12.52)  (p = 0.164) | 0.93 (0.22 to 3.87)  (p = 0.919) | 0.69 (0.08 to 7.35)  (p = 0.743) | 0.08 (0.01 to 0.69)  (p = 0.031) | 0.68 (0.10 to 6.11)  (p = 0.703) | 1.33 (0.31 to 5.51)  (p = 0.691) |
| **4. Dissatisfied climbing stairs** | | | | | | | | | | |
|  | 0.106 | 1.79 (0.63 to 5.31)  (p = 0.279) | 0.55 (0.17 to 1.74) (p = 0.306) | 0.69 (0.19 to 2.62) (p = 0.583) | 0.29 (0.07 to 1.04)  (p = 0.072) | 3.77 (1.08 to 15.77) (p = 0.048) | 0.58 (0.08 to 3.52)  (p = 0.558) | 2.85 (0.37 to 22.13) (p = 0.303) | 0.77 (0.13 to 3.86)  (p = 0.758) | 1.03 (0.28 to 4.06)  (p = 0.967) |
| **5. Dissatisfied making a tight fist*** | | | | | | | | | | |
| **6. Dissatisfied getting in and out of a bathtub or shower** | | | | | | | | | | |
|  | 0.178 | 1.34 (0.43 to 4.25)  (p = 0.612) | 0.08 (0.01 to 0.50) (p = 0.025) | 1.05 (0.21 to 4.78) (p = 0.949) | 0.54 (0.13 to 2.02)  (p = 0.369) | 4.19 (1.08 to 19.04) (p = 0.048) | 1.26 (0.12 to 29.27) (p = 0.858) | 1.40 (0.16 to 15.74) (p = 0.766) | 1.59 (0.19 to 33.87)  (p = 0.699) | 1.01 (0.25 to 3.76)  (p = 0.991) |
| **7. Problems getting comfortable to sleep** | | | | | | | | | | |
|  | 0.199 | 1.40 (0.48 to 4.08)  (p = 0.536) | 0.45 (0.11 to 1.59) (p = 0.228) | 0.51 (0.11 to 2.04) (p = 0.357) | 0.54 (0.14 to 1.90)  (p = 0.351) | 4.45 (1.28 to 17.80) (p = 0.024) | 4.88 (0.57 to 108.70) (p = 0.198) | 0.61 (0.07 to 4.56)  (p = 0.631) | 3.49 (0.48 to 71.89)  (p = 0.283) | 2.16 (0.57 to 8.65)  (p = 0.260) |
| **8. Problems bending down or kneeling** | | | | | | | | | | |
|  | 0.283 | 3.91 (0.87 to 23.43)  (p = 0.097) | 0.11 (0.02 to 0.51) (p = 0.008) | 2.14 (0.33 to 19.50)  (p = 0.457) | 0.34 (0.05 to 1.79)  (p = 0.222) | 10.03 (1.91 to 75.10)  (p = 0.012) | 0.07 (0.01 to 1.00)  (p = 0.087) | 1.08 (0.03 to 18.04) (p = 0.960) | 0.15 (0.01 to 1.68)  (p = 0.187) | 0.68 (0.10 to 6.19)  (p = 0.708) |
| **9. Problems using buttons, snaps, hooks, or zippers** | | | | | | | | | | |
|  | 0.181 | 2.38 (0.30 to 26.96)  (p = 0.426) | 0.16 (0.01 to 1.79) (p = 0.205) | 0.38 (0.01 to 6.61) (p = 0.559) | 2.65 (0.19 to 85.79)  (p = 0.507) | 3.29 (0.24 to 111.50)  (p = 0.421) | 0.24 (0.01 to 7.23)  (p = 0.354) | 0.03 (0.01 to 0.62)  (p = 0.035) | 0.08 (0.01 to 2.44)  (p = 0.109) | 0.67 (0.03 to 6.94)  (p = 0.763) |
| **10. Problems cutting your own fingernails** | | | | | | | | | | |
|  | 0.053 | 0.80 (0.26 to 2.33)  (p = 0.686) | 1.00 (0.28 to 3.32) (p = 1.000) | 0.92 (0.22 to 3.47) (p = 0.905) | 1.84 (0.52 to 6.92)  (p = 0.349) | 1.78 (0.49 to 6.76)  (p = 0.382) | 0.91 (0.13 to 8.30)  (p = 0.926) | 0.34 (0.05 to 2.45)  (p = 0.269) | 0.95 (0.16 to 7.59)  (p = 0.953) | 1.17 (0.30 to 4.20)  (p = 0.814) |
| **11. Problems dressing yourself** | | | | | | | | | | |
|  | 0.128 | 2.15 (0.71 to 6.77)  (p = 0.179) | 1.06 (0.27 to 3.87) (p = 0.933) | 0.85 (0.19 to 3.21) (p = 0.788) | 0.93 (0.23 to 3.75)  (p = 0.920) | 1.93 (0.50 to 8.07)  (p = 0.349) | 0.30 (0.03 to 2.54)  (p = 0.262) | 0.28 (0.04 to 2.00)  (p = 0.203) | 0.59 (0.10 to 3.74)  (p = 0.548) | 2.38 (0.64 to 8.78)  (p = 0.188) |
| **12. Problems walking** | | | | | | | | | | |
|  | 0.182 | 3.16 (1.12 to 9.51)  (p = 0.034) | 0.23 (0.05 to 0.81) (p = 0.031) | 0.63 (0.15 to 2.47) (p = 0.511) | 1.17 (0.34 to 4.07)  (p = 0.800) | 1.45 (0.42 to 5.04)  (p = 0.550) | 0.40 (0.06 to 2.64)  (p = 0.336) | 0.45 (0.05 to 3.26)  (p = 0.432) | 0.47 (0.08 to 2.63)  (p = 0.383) | 2.19 (0.62 to 8.29)  (p = 0.228) |
| **13. Problems moving after you have been sitting or lying down** | | | | | | | | | | |
|  | 0.281 | 3.38 (0.95 to 13.47)  (p = 0.068) | 0.33 (0.05 to 1.58) (p = 0.192) | 1.28 (0.24 to 6.57) (p = 0.768) | 1.88 (0.41 to 9.49)  (p = 0.424) | 7.13 (1.28 to 63.99) (p = 0.041) | 0.14 (0.01 to 1.90)  (p = 0.137) | 1.04 (0.10 to 14.87) (p = 0.974) | 0.49 (0.06 to 4.70)  (p = 0.503) | 2.76 (0.65 to 12.16)  (p = 0.167) |
| **14. Problems going out by yourself** | | | | | | | | | | |
|  | 0.192 | 3.04 (1.07 to 9.26)  (p = 0.042) | 0.36 (0.10 to 1.20) (p = 0.107) | 0.49 (0.11 to 1.92) (p = 0.313) | 0.94 (0.26 to 3.27)  (p = 0.926) | 2.14 (0.64 to 7.52)  (p = 0.223) | 0.26 (0.03 to 1.72)  (p = 0.178) | 0.81 (0.10 to 5.97)  (p = 0.840) | 0.26 (0.04 to 1.41)  (p = 0.135) | 3.20 (0.86 to 14.36)  (p = 0.099) |
| **15. Problems driving** | | | | | | | | | | |
|  | 0.181 | 2.12 (0.72 to 6.45)  (p = 0.176) | 0.65 (0.16 to 2.37) (p = 0.522) | 1.42 (0.34 to 5.76) (p = 0.624) | 2.23 (0.62 to 8.58)  (p = 0.226) | 2.15 (0.58 to 8.64)  (p = 0.258) | 2.23 (0.23 to 51.86) (p = 0.529) | 0.66 (0.09 to 5.50)  (p = 0.682) | 2.84 (0.37 to 60.04)  (p = 0.378) | 2.32 (0.61 to 8.97)  (p = 0.211) |
| **16. Problems cleaning yourself after going to the bathroom*** | | | | | | | | | | |
| **17. Problems turning knobs or levers*** | | | | | | | | | | |
|  | | | | | | | | | | |
| **18. Problems with writing or typing*** | | | | | | | | | | |
|  | | | | | | | | | | |
| **19. Problems with pivoting** | | | | | | | | | | |
|  | 0.216 | 4.42 (1.21 to 20.00)  (p = 0.034) | 0.28 (0.07 to 1.06) (p = 0.064) | 1.82 (0.39 to 10.84)  (p = 0.472) | 0.41 (0.08 to 1.82)  (p = 0.262) | 5.82 (1.41 to 29.89) (p = 0.021) | 0.48 (0.04 to 4.32)  (p = 0.531) | 3.22 (0.27 to 34.04) (p = 0.329) | 0.40 (0.04 to 2.67)  (p = 0.382) | 0.49 (0.10 to 2.47)  (p = 0.377) |
| **20. Problems doing usual physical recreational activities such as bicycling, jogging, or walking** | | | | | | | | | | |
|  | 0.169 | 1.64 (0.43 to 6.80)  (p = 0.473) | 0.42 (0.10 to 1.76) (p = 0.231) | 2.95 (0.49 to 30.68)  (p = 0.289) | 0.24 (0.04 to 1.22)  (p = 0.103) | 9.77 (2.05 to 64.14) (p = 0.008) | 0.69 (0.06 to 5.84)  (p = 0.742) | 6.79 (0.57 to 83.66) (p = 0.118) | 0.94 (0.10 to 6.11)  (p = 0.954) | 1.19 (0.21 to 9.75)  (p = 0.857) |
| **21. Problems doing usual leisure activities, such as hobbies, crafts, gardening, card-playing, or going out with friends** | | | | | | | | | | |
|  | 0.287 | 1.25 (0.41 to 3.79)  (p = 0.696) | 0.48 (0.12 to 1.73) (p = 0.274) | 0.75 (0.17 to 3.20) (p = 0.704) | 0.64 (0.15 to 2.40)  (p = 0.517) | 10.92 (2.80 to 53.49)  (p = 0.001) | 0.40 (0.05 to 3.15)  (p = 0.374) | 0.66 (0.07 to 5.60)  (p = 0.711) | 0.75 (0.12 to 4.89)  (p = 0.756) | 3.75 (0.96 to 16.79)  (p = 0.065) |
| **22. Problems with sexual activity** | | | | | | | | | | |
|  | 0.434 | 6.71 (1.92 to 27.93)  (p = 0.005) | 0.46 (0.09 to 2.12) (p = 0.333) | 2.30 (0.42 to 14.14)  (p = 0.342) | 0.56 (0.11 to 2.61)  (p = 0.470) | 26.04 (4.60 to 250.9)  (p = 0.001) | 0.90 (0.08 to 13.21) (p = 0.937) | 0.97 (0.08 to 13.64) (p = 0.983) | 1.18 (0.13 to 15.13)  (p = 0.887) | 2.26 (0.47 to 12.01)  (p = 0.316) |
| **23. Problems with light housework or yard work, such as dusting, washing dishes, or watering plants*** | | | | | | | | | | |
| **24. Problems with doing heavy housework or yard work, such as washing floors, vacuuming, or moving lawns** | | | | | | | | | | |
|  | 0.182 | 1.94 (0.63 to 6.37)  (p = 0.258) | 0.38 (0.10 to 1.33) (p = 0.135) | 4.94 (0.98 to 40.41)  (p = 0.082) | 0.25 (0.05 to 1.03)  (p = 0.072) | 4.72 (1.21 to 24.20) (p = 0.037) | 1.33 (0.20 to 9.12)  (p = 0.766) | 1.11 (0.05 to 11.36) (p = 0.937) | 1.47 (0.26 to 8.13)  (p = 0.653) | 1.48 (0.32 to 8.55)  (p = 0.632) |
| **25. Problems doing usual work, such as a paid job, housework, or volunteer activity** | | | | | | | | | | |
|  | 0.120 | 2.47 (0.91 to 6.97)  (p = 0.079) | 0.54 (0.16 to 1.71) (p = 0.302) | 0.81 (0.22 to 2.96) (p = 0.753) | 0.84 (0.25 to 2.78)  (p = 0.779) | 1.13 (0.34 to 3.69)  (p = 0.841) | 0.65 (0.11 to 4.17)  (p = 0.645) | 0.42 (0.05 to 2.90)  (p = 0.396) | 1.07 (0.21 to 5.63)  (p = 0.939) | 2.37 (0.69 to 9.04)  (p = 0.181) |
| **Difficulties at Daily Living / Emotional Function** | | | | | | | | | | |
| **26. Limp while walking** | | | | | | | | | | |
|  | 0.176 | 2.24 (0.69 to 7.92)  (p = 0.190) | 0.58 (0.16 to 2.05) (p = 0.386) | 0.61 (0.14 to 2.86) (p = 0.515) | 0.54 (0.11 to 2.19)  (p = 0.399) | 3.52 (0.91 to 15.58) (p = 0.077) | 0.32 (0.03 to 2.28)  (p = 0.280) | 1.81 (0.17 to 16.66) (p = 0.602) | 0.67 (0.08 to 4.00)  (p = 0.681) | 6.01 (0.97 to 117.70) (p = 0.107) |
| **27. Avoiding using painful limb(s) or back** | | | | | | | | | | |
|  | 0.263 | 1.47 (0.44 to 5.09)  (p = 0.535) | 0.27 (0.07 to 0.97) (p = 0.050) | 0.18 (0.03 to 0.78) (p = 0.028) | 0.47 (0.10 to 1.96)  (p = 0.320) | 7.68 (1.90 to 38.97) (p = 0.007) | 0.18 (0.01 to 1.55)  (p = 0.146) | 1.77 (0.18 to 16.27) (p = 0.611) | 0.23 (0.02 to 1.60)  (p = 0.175) | 2.63 (0.56 to 16.46)  (p = 0.250) |
| **28. Leg locks or gives way** | | | | | | | | | | |
|  | 0.263 | 2.32 (0.77 to 7.344)  (p = 0.138) | 0.28 (0.07 to 0.98) (p = 0.055) | 0.57 (0.12 to 2.48) (p = 0.447) | 0.76 (0.19 to 2.79)  (p = 0.681) | 4.44 (1.25 to 17.77) (p = 0.026) | 0.18 (0.02 to 1.26)  (p = 0.098) | 0.14 (0.01 to 1.69)  (p = 0.175) | 0.30 (0.04 to 1.69)  (p = 0.194) | 2.80 (0.67 to 15.13)  (p = 0.184) |
| **29. Problems with concentration** | | | | | | | | | | |
|  | 0.094 | 1.07 (0.32 to 3.53)  (p = 0.914) | 0.80 (0.18 to 3.03) (p = 0.754) | 1.41 (0.31 to 5.87) (p = 0.640) | 1.21 (0.29 to 5.48)  (p = 0.800) | 2.85 (0.65 to 14.76) (p = 0.180) | 0.34 (0.05 to 2.60)  (p = 0.288) | 0.29 (0.04 to 2.26)  (p = 0.222) | 0.25 (0.04 to 1.65)  (p = 0.136) | 1.02 (0.22 to 4.00)  (p = 0.982) |
| **30. Doing too much in one day affects what you do the next day** | | | | | | | | | | |
|  | 0.223 | 2.15 (0.69 to 7.03)  (p = 0.191) | 0.35 (0.09 to 1.24) (p = 0.110) | 0.44 (0.10 to 1.90) (p = 0.273) | 0.34 (0.07 to 1.31)  (p = 0.136) | 4.78 (1.27 to 21.77) (p = 0.028) | 0.09 (0.01 to 0.89)  (p = 0.068) | 1.03 (0.08 to 9.48)  (p = 0.982) | 0.12 (0.01 to 0.92)  (p = 0.080) | 2.10 (0.48 to 11.95)  (p = 0.353) |
| **31. Act irritable toward those around them*** | | | | | | | | | | |
| **32. Feels tired** | | | | | | | | | | |
|  | 0.150 | 1.83 (0.65 to 5.23)  (p = 0.253) | 0.33 (0.09 to 1.08) (p = 0.076) | 1.47 (0.39 to 6.01) (p = 0.574) | 1.45 (0.42 to 4.98)  (p = 0.547) | 1.93 (0.59 to 6.43)  (p = 0.275) | 0.27 (0.04 to 1.70)  (p = 0.179) | 0.41 (0.04 to 2.98)  (p = 0.394) | 0.30 (0.05 to 1.56)  (p = 0.172) | 2.00 (0.55 to 8.47)  (p = 0.309) |
| **33. Feels disabled** | | | | | | | | | | |
|  | 0.282 | 1.41 (0.42 to 4.82)  (p = 0.577) | 0.28 (0.07 to 1.00) (p = 0.056) | 0.57 (0.12 to 2.79) (p = 0.479) | 0.43 (0.08 to 1.89)  (p = 0.292) | 10.82 (2.55 to 63.77) (p = 0.003) | 0.75 (0.10 to 5.42)  (p = 0.774) | 0.28 (0.01 to 3.90)  (p = 0.387) | 0.90 (0.14 to 5.13)  (p = 0.904) | 3.75 (0.71 to 30.45)  (p = 0.153) |
| **34. Feel angry or frustrated that they have this injury or arthritis** | | | | | | | | | | |
|  | 0.126 | 0.98 (0.35 to 2.73)  (p = 0.970) | 0.32 (0.09 to 1.03) (p = 0.063) | 0.62 (0.16 to 2.27) (p = 0.474) | 1.24 (0.37 to 4.09)  (p = 0.718) | 2.68 (0.83 to 9.08)  (p = 0.102) | 0.82 (0.13 to 5.21)  (p = 0.833) | 0.27 (0.03 to 1.89)  (p = 0.210) | 0.70 (0.13 to 3.71)  (p = 0.674) | 0.82 (0.23 to 2.90)  (p = 0.751) |
| **Bothersome Index** | | | | | | | | | | |
| **35. Problems using your hands, arms, or legs** | | | | | | | | | | |
|  | 0.099 | 1.32 (0.48 to 3.56)  (p = 0.589) | 0.66 (0.20 to 2.05) (p = 0.475) | 0.62 (0.17 to 2.21) (p = 0.470) | 1.27 (0.39 to 4.09)  (p = 0.686) | 2.63 (0.83 to 8.78)  (p = 0.104) | 0.46 (0.07 to 2.81)  (p = 0.395) | 1.07 (0.17 to 7.53)  (p = 0.944) | 0.78 (0.15 to 3.99)  (p = 0.756) | 1.46 (0.44 to 5.04)  (p = 0.539) |
| **36. Problems using your back** | | | | | | | | | | |
|  | 0.149 | 1.37 (0.48 to 3.92)  (p = 0.553) | 0.29 (0.07 to 1.03) (p = 0.071) | 1.05 (0.26 to 4.13) (p = 0.943) | 1.05 (0.30 to 3.58)  (p = 0.940) | 3.05 (0.90 to 11.27) (p = 0.080) | 0.67 (0.09 to 6.26)  (p = 0.706) | 0.89 (0.12 to 6.96)  (p = 0.906) | 1.37 (0.23 to 10.87)  (p = 0.739) | 1.31 (0.37 to 4.59)  (p = 0.677) |
| **37. Problems doing work around your home** | | | | | | | | | | |
|  | 0.170 | 3.51 (1.17 to 11.41)  (p = 0.029) | 0.43 (0.09 to 1.65) (p = 0.241) | 0.40 (0.07 to 1.81) (p = 0.261) | 1.08 (0.28 to 4.12)  (p = 0.906) | 3.15 (0.80 to 14.20) (p = 0.112) | 0.47 (0.05 to 4.83)  (p = 0.494) | 1.17 (0.16 to 11.38) (p = 0.881) | 1.01 (0.16 to 8.54)  (p = 0.992) | 1.37 (0.35 to 5.12)  (p = 0.643) |
| **38. Problems with bathing, dressing, toileting, or other personal care** | | | | | | | | | | |
|  | 0.124 | 2.47 (0.89 to 7.20)  (p = 0.089) | 0.88 (0.25 to 2.95) (p = 0.838) | 0.48 (0.11 to 1.78) (p = 0.289) | 0.31 (0.08 to 1.06)  (p = 0.071) | 2.30 (0.69 to 8.38)  (p = 0.187) | 1.47 (0.21 to 14.03) (p = 0.712) | 0.95 (0.13 to 6.35)  (p = 0.957) | 2.81 (0.52 to 22.82)  (p = 0.266) | 1.09 (0.30 to 3.85)  (p = 0.896) |
| **39. Problems with sleep and rest*** | | | | | | | | | | |
|  | | | | | | | | | | |
| **40. Problems with leisure or recreational activities** | | | | | | | | | | |
|  | 0.149 | 2.90 (1.04 to 8.37)  (p = 0.044) | 0.44 (0.12 to 1.49) (p = 0.202) | 1.24 (0.31 to 4.79) (p = 0.756) | 1.46 (0.41 to 5.18)  (p = 0.556) | 2.37 (0.68 to 8.83)  (p = 0.181) | 0.58 (0.08 to 4.23)  (p = 0.576) | 0.86 (0.13 to 6.46)  (p = 0.878) | 0.72 (0.13 to 4.48)  (p = 0.712) | 0.69 (0.18 to 2.48)  (p = 0.581) |
| **41. Problems with friends, family, or other important people in your life*** | | | | | | | | | | |
|  | | | | | | | | | | |
| **42. Problems with thinking, concentrating, or remembering** | | | | | | | | | | |
|  | 0.182 | 0.41 (0.10 to 1.44)  (p = 0.180) | 0.09 (0.01 to 0.61) (p = 0.040) | 1.95 (0.40 to 9.45) (p = 0.402) | 0.86 (0.19 to 3.75)  (p = 0.838) | 4.00 (0.90 to 21.79) (p = 0.083) | 0.15 (0.01 to 1.80)  (p = 0.128) | 0.60 (0.06 to 7.05)  (p = 0.668) | 0.37 (0.04 to 3.50)  (p = 0.354) | 1.09 (0.25 to 4.30)  (p = 0.903) |
| **43. Problems adjusting or coping with injury or arthritis** | | | | | | | | | | |
|  | 0.113 | 1.63 (0.60 to 4.54)  (p = 0.340) | 0.31 (0.08 to 1.03) (p = 0.068) | 2.51 (0.70 to 9.50) (p = 0.160) | 1.20 (0.35 to 4.09)  (p = 0.768) | 1.35 (0.41 to 4.50)  (p = 0.622) | 1.80 (0.30 to 12.22) (p = 0.525) | 0.52 (0.07 to 3.43)  (p = 0.494) | 0.71 (0.13 to 4.20)  (p = 0.688) | 0.75 (0.20 to 2.60)  (p = 0.655) |
| **44. Problems doing usual work** | | | | | | | | | | |
|  | 0.094 | 1.62 (0.60 to 4.48)  (p = 0.342) | 0.42 (0.13 to 1.29) (p = 0.136) | 1.20 (0.34 to 4.39) (p = 0.777) | 1.45 (0.44 to 4.76)  (p = 0.536) | 1.76 (0.56 to 5.70)  (p = 0.335) | 0.37 (0.05 to 2.22)  (p = 0.289) | 1.37 (0.21 to 9.70)  (p = 0.741) | 0.30 (0.05 to 1.50)  (p = 0.161) | 0.89 (0.26 to 3.07)  (p = 0.850) |
| **45. Problems with feeling dependent on others** | | | | | | | | | | |
|  | 0.192 | 1.01 (0.33 to 3.02)  (p = 0.989) | 0.15 (0.02 to 0.67) (p = 0.023) | 1.12 (0.25 to 4.67) (p = 0.879) | 1.50 (0.41 to 5.61)  (p = 0.536) | 2.28 (0.64 to 8.75)  (p = 0.210) | 1.69 (0.23 to 15.71) (p = 0.614) | 0.17 (0.02 to 1.29)  (p = 0.094) | 0.51 (0.07 to 4.49)  (p = 0.510) | 2.0 (0.54 to 7.52)  (p = 0.295) |
| **46. Problems with stiffness or pain** | | | | | | | | | | |
|  | 0.159 | 1.01 (0.35 to 2.84)  (p = 0.986) | 0.31 (0.09 to 1.03) (p = 0.064) | 0.36 (0.08 to 1.37) (p = 0.148) | 0.94 (0.27 to 3.10)  (p = 0.924) | 3.47 (1.06 to 12.41) (p = 0.045) | 0.42 (0.06 to 2.75)  (p = 0.371) | 0.16 (0.01 to 1.28)  (p = 0.106) | 0.53 (0.09 to 2.89)  (p = 0.460) | 0.57 (0.15 to 1.98)  (p = 0.377) |

*Could not fit a logistic regression because one or more of the predictors are linearly dependent.
